# The Vacant Secretaryship at Norwich: Hundreds of Applications or None?

**Published:** 1915-10-16

**Authors:** 


### THE EDITOR'S LETTER-BOX.

### THE VACANT SECRETARYSHIP AT NORWICH.

### Hundreds of Applications or None?

*To the Editor of* The Hospital.

Sir,—You have very properly intimated that the appointment of Mr. Hazell to the superintendency of the Manchester Royal Infirmary is a suitable and satisfactory one. May I take the liberty of pointing out that the Board of the Norfolk and Norwich Hospital, whose secretary has been chosen to undertake one of the most responsible administrative posts in the hospitals of the United Kingdom, are seeking to replace his long service, proved record, and valuable experience by a successor whose remuneration is fixed at £250 per annum, whose age must be between thirty and fifty, and who must have had experience of hospital management? I gather from your columns that the present bed accommodation of the hospital is about 500, from 250 of which the Board are receiving a grant from the War Office on a scale which will doubtless secure the institution against actual loss. From every point of view, therefore, it must be realised that the present is a singularly inauspicious moment to attempt to save money by reducing the salary attached to the office of the principal administrator. As a hospital superintendent of many years' standing, I submit that no self-respecting men possessed of experience and grit can apply for the position and at the same time retain their dignity without making their applications conditional that after a three months' trial the successful candidate shall enjoy an increase of salary to £400 to £500 per annum from the commencement of his service. A post which entails responsibility of the kind which the secretaryship of the Norfolk and Norwich Hospital—the pattern hospital of East Anglia —must do, should be filled by a man of keen intellect, sound judgment, and of educational standing. Such a man commands remuneration according to merit, and it is "up to" Norwich, with its prosperous industries, to work to the standard, and not down to a level of remuneration for their principal administrator so low as to beggar description.—Yours faithfully,

Oct. 11, 1915. Hospital Superintendent.

